# Intensified Springtime Deep Convection over the South China Sea and the Philippine Sea Dries Southern China

**DOI:** 10.1038/srep30470

**Published:** 2016-07-27

**Authors:** Zhenning Li, Song Yang, Bian He, Chundi Hu

**Affiliations:** 1 School of Atmospheric Sciences, Sun Yat-sen University, Guangzhou, Guangdong, China; 2 Guangdong Province Key Laboratory for Climate Change and Natural Disaster Studies, Sun Yat-sen University, Guangzhou, Guangdong, China; 3 Institute of Earth Climate and Environment System, Sun Yat-sen University, Guangzhou, Guangdong, China; 4 State Key Laboratory of Numerical Modeling for Atmospheric Sciences and Geophysical Fluid Dynamics, Institute of Atmospheric Physics, Chinese Academy of Sciences, Beijing, China; 5 School of Atmospheric Sciences, Nanjing University, Nanjing, Jiangsu, China

## Abstract

Springtime rainfall, accounting for 25–40% of the annual rainfall in southern China, exerts great agricultural and socioeconomic impacts on the region. In the recent decades, southern China has experienced a significant declining trend of precipitation in boreal spring. Meanwhile, precipitation has increased over the South China Sea and the Philippine Sea (SCS-PhS). This paper presents observational and modeling evidences suggesting that the intensified latent heating released by the convection over SCS-PhS leads to suppressed springtime rainfall over southern China. Moisture budget analysis indicates that the drying trend over southern China is due mainly to weakened convergence of moisture flux, which is controlled by a heat-induced anomalous overturning circulation reinforced by the convection over SCS-PhS. Further idealized simulations support the feature that the heat-induced overturning circulation and its corresponding anomalous cyclone can be well established in several days under the spring mean flow condition. Thus, this rapid dynamic process is associated with both the intraseasonal-to-interannual variations and the long-term change of the springtime rainfall over southern China.

What makes the Asian monsoon system distinguished from other monsoon regimes over the world is its abrupt seasonal transition and associated stepwise evolution within the rainy season^1^,^2^, besides the intensity of the regional monsoon. Since the rainy-season precipitation of the Asian monsoon system exerts tremendous socioeconomic impacts on many places inside and outside Asia, the variations and their effects of the summer monsoon rainfall have been investigated extensively^3^,^4^,^5^. However, the springtime rainfall over southern China (SRSC) from mid-March to pre-Meiyu in May, the major rainfall of the early rainy season in East Asia, has been studied less comprehensively. Near the mountainous border between Guangdong Province and Fujian Province of China, the rainfall of boreal spring accounts for 25–40% of the annual total precipitation^1^,^6^ (illustrated in Fig. 1a). The anomalies of springtime rainfall often lead to severe droughts or floods and thus serious damage in southern China. Revealing the variation of SRSC and the responsible mechanism is essential because such knowledge contributes to improved weather and climate forecasts and even benefits sustainable social development.

Previous studies have shown that SRSC is related to the development of a low-pressure system over southwestern China and the western North Pacific subtropical high^5^. Documented research has also suggested that the dynamic and thermodynamic processes of the Tibetan Plateau^7^ and the sea surface temperature (SST) over the western Pacific^8^ influence the variability of SRSC. However, previous studies have been mainly focused on the interannual variability and prediction of SRSC^9^, but how SRSC behaves in the long-term climate change context is still unrevealed.

There are still many open questions regarding the long-term variations of monsoon rainfall. Although focusing on summertime rainfall, recent studies have shown that elevated aerosols can affect the regional monsoon water cycle via altering energy balance and modulating the microphysics of cloud and rainfall^10^,^11^,^12^. For example, the observed precipitation decreasing trend over South Asia can be attributed mainly to human-influenced aerosol emissions^13^, and the positive feedback in the aerosol-cloud-precipitation interaction may aggravate drought in Asian arid and semiarid regions^14^. Furthermore, for both interannual variability and long-term change, the relationship between tropical SST and the Asian summer monsoon reflects an atmospheric response to the latent heat release of tropical deep convection^15^, and the role of atmospheric heating in modulating large-scale atmospheric dynamics has long been an important issue to address in climate research^16^,^17^,^18^. For instance, the dynamical feedback within the broader Asian monsoon system dries South Asia by the change in atmospheric circulation associated with rising SST and convection heating over the tropical western Pacific^19^,^20^. Moreover, recent studies suggested that the SST threshold for triggering tropical deep convection, which associated heating serves as a direct driver for tropical atmospheric circulation, has risen in the warming climate, meaning that the relationship between SST and *in-situ* convection may have changed in the long-term climate context^21^.

Considering the inherent uncertainty of the aerosol-cloud-precipitation interaction, the SST-convection relationship, the complicated air-sea-land interaction processes in the Asian monsoon region, and the model biases in simulating convection, the current study is aimed to obtain the change in convective heat source directly from observation/reanalysis data (verified by various data sets) over the South China Sea and the Philippine Sea (SCS-PhS) and unravel its role in driving the atmospheric circulation that in turn contributes to changes in the SRSC. Results obtained potentially improve the prediction and projection of the change in SRSC.

## Results

### Observed intensified deep convection over SCS-PhS and drying trend over southern China. 

**Figure 1:**
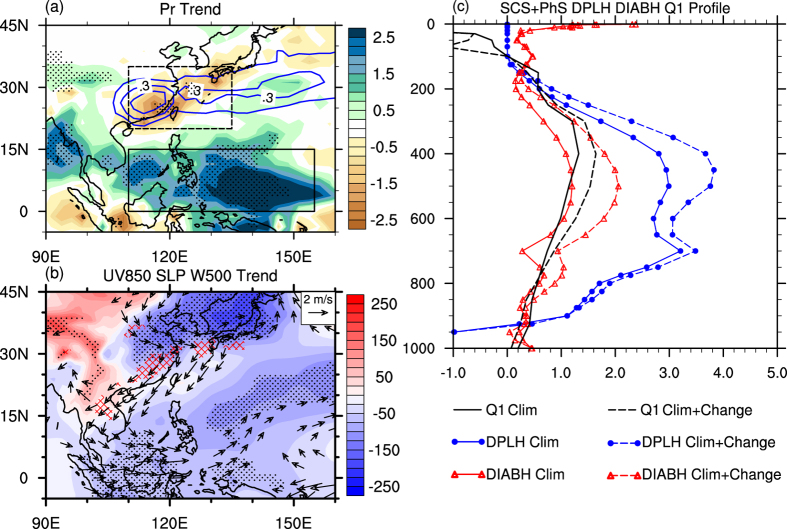
(**a**) Shading areas show the linear trend of MAM precipitation [mm day^−1^ (35yr)^−1^] for 1979–2013. Areas of >90% confidence level are marked by black dots. Blue contours at 5% intervals show the areas where the ratio of climatological MAM precipitation to climatological annual precipitation exceeds 25%. (**b**) Shading and dotted areas are the same as (**a**), but for sea level pressure [Pa (35yr)^−1^]. Vectors denote the linear trend of 850-hPa wind and hatching indicates where the linear trend of 500-hPa vertical velocity is above 0.025 [Pa s^−1^ (35yr)^−1^]. Both are statistically significant at the 90% confidence level. (**c**) MAM climatology, and climatology plus 35-yr linear change, of heating profile averaged over the lower box in (**a**). Black lines are for the Q1 derived from the NCEP-DOE Reanalysis. Red and blue lines are for diabetic heating and deep-convection latent heating from the NCEP CFS 1-hour forecasting data. This figure was generated by NCL version 6.3.0: http://www.ncl.ucar.edu/ and was additionally paneled by licensed Adobe Illustrator CS6 version 16.0.0.

As shown by the blue contours in Fig. 1a, climatologically the ratio of March-April-May (MAM) precipitation to annual precipitation exceeds 25% in southern China, Taiwan, and southern Japan. Near the mountainous border between Guangdong Province and Fujian Province of China, the ratio even reaches 40%. A noted feature, however, is that the springtime rainfall over southern China, Taiwan, and southern Japan has decreased significantly in the past 35 years (1979–2013). The most obvious decrease occurs over the inland and coastal southern China, exhibiting a negative trend of more than 2.0 [mm day^−1^ (35yr)^−1^] or a nearly 50% decrease in the climatological MAM rainfall in the last 35 years. Meanwhile, springtime rainfall exhibits a positive trend near the west coast of the Indo-China Peninsula, the southern South China Sea, and the southern Philippine Sea. A specific region centered near 140°E/5°N, with a spatial extent of approximately 10° in zonal and 5° in meridional directions, exhibits the largest change (Fig. 1a). The positive trend of springtime rainfall is above 2.5 [mm day^−1^ (35yr)^−1^], accounting for more than 40% of the 35-year climatological MAM mean rainfall of the region.

To reveal the associated changes in atmospheric circulation, we show in Fig. 1b the trends of spring sea level pressure (SLP), 500-hPa vertical velocity, and 850-hPa winds. A pronounced feature is that SLP increases over inland continental Asia and decreases over eastern China and the western Pacific Ocean, corresponding to the low-level northerly anomalies along coastal East Asia. The linear trends of 500-hPa vertical velocity are above 0.025 [Pa s^−1^ (35yr)^−1^] to the north of 10°N and anomalous descending motions are seen over northern Vietnam, southern China, and southern Japan (see red hatching). It is interesting to note that the change in 850-hPa winds exhibits a wave train pattern in which an anomalous cyclone is located over SCS-PhS. This feature seems to be a Gill-type response to the strengthened deep convection over the SCS-PhS^22^ region.

Based on the above analysis, we further estimate the change in convection heating over SCS-PhS (the lower box in Fig. 1a) in the last 35 years. In Fig. 1c, the solid lines depict MAM climatological rates of deep convection heating, atmospheric diabatic heating, and Q_1_ (see Methods), respectively. The dashed lines show the climatology plus the 35-yr linear changes of those variables. It can be found that, in the free troposphere from 800 hPa to 200 hPa, both diabatic heating and deep convection heating increase. The most significant change in latent heating appears from 600 hPa to 300 hPa, with the maximum increase in heating approaching +0.9 K day^−1^ near 450 hPa. Moreover, the profile of change in diabatic heating is very similar to that in latent heating, indicating that the intensification of deep convection heating dominates the change in diabatic heating over SCS-PhS. Although its value is relatively smaller, Q_1_ exhibits a similar intensified pattern in the free troposphere, which verifies the robustness of the strengthened convection heating over SCS-PhS.

Despite that convection heating is always hard to obtain, especially over oceans, the trend and amplitude of the CMAP and GPCP data sets are overall consistent with each other over SCS-PhS (see Supplementary Information Fig. S1a,b). Moreover, the decreased outgoing longwave radiation (OLR, Fig. S1c) also indicates deeper convection. Note that the lower OLR may be induced by biases in the satellite product due to increasing cloud overlap caused by higher SST^23^,^24^ (Fig. S2). Nevertheless, the drop in SLP, the cyclonic response in low-level winds (Fig. 1b), and the increasing trend in the CAPE (convective available potential energy, Fig. S1d) all consistently imply that the convection over the SCS-PhS region has become stronger in the recent decades. Thus, observations point to a change in the springtime circulation that has resulted in more rainfall over SCS-PhS and less rainfall over southern China.

**Figure 2:**
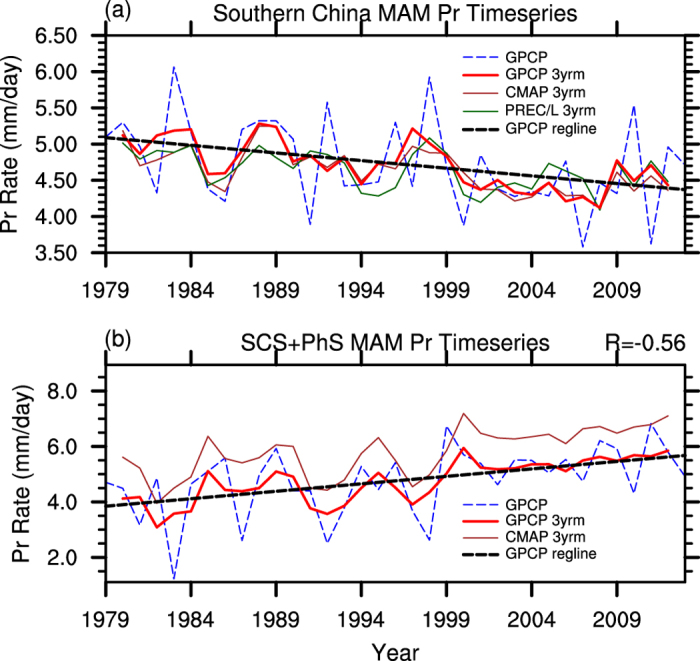
(**a**) 3-year running mean applied on area-weighted MAM mean precipitation in the upper box in Fig. 1a from three different data sets: GPCP (thick red line), CMAP (brown line), and PREC/L (green line, only for precipitation over land). Blue dashed line is the original area-weighted MAM mean precipitation from GPCP and black dashed line is the linear trend derived from GPCP. (**b**) Same as (**a**) but for the lower box in Fig. 1a. The value shown at the upper right corner of (**b**) indicates the coefficient of correlation between the detrended area-weighted MAM mean precipitation (PREC/L) in the upper box over land and the detrended area-weighted MAM mean precipitation (GPCP) in the lower box. This figure was generated by NCL version 6.3.0: http://www.ncl.ucar.edu/.

To depict the potential relationship of changes in springtime rainfall between SCS-PhS and southern China, we show in Fig. 2 the area-weighted MAM mean precipitation for the two boxes in Fig. 1a from three different data sets. For the period of 1979–2013, the MAM mean precipitation over southern China decreases, with a steady decline in 3-year running mean values, as shown for the upper box of Fig. 1a. The drying tendency in this region is robust among all the three data sets analyzed, including the gauge-based reconstructed precipitation data over land (PREC/L).

On the other hand, the MAM mean precipitation increases over SCS-PhS (Fig. 2b). Although the CMAP and GPCP data sets show some differences (Fig. 2b), their trends and amplitudes are consistent with each other. On interannual time scales, the detrended springtime rainfall over SCS-PhS is highly correlated with that over southern China (R = −0.56 for 1979–2013, significant at the 99.9% confidence level). Then, does this relationship also contribute to the long-term drying trend in southern China? If yes, what is the physical mechanism and how is it compared with the summertime Pacific-Japan (P-J) teleconection relationship^25^? Model simulations are helpful for addressing these questions.

### Modeled heat-induced overturning circulation that dries southern China. 

Several numerical experiments are performed based on the Community Earth System Model version 1.2.2 (CESM1.2.2), in which intensified convection heating is imposed in fully-coupled simulations and idealized heating profile based on observation is introduced to idealized AGCM simulations (see Methods). We first examine the result from the fully-coupled CESM experiments.

**Figure 3:**
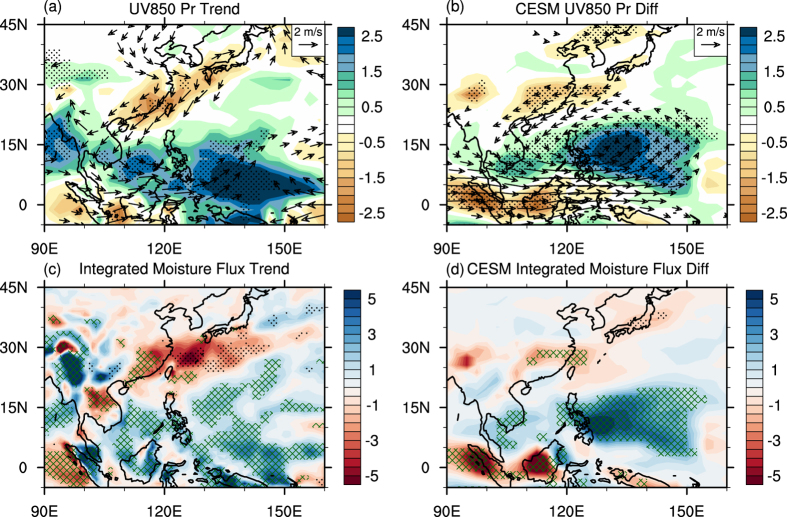
(**a**) Shading areas show the linear trend of MAM precipitation [mm day^−1^ (35yr)^−1^] for 1979–2013 with areas of >90% confidence level marked by black dots. Vectors denote the linear trend of 850-hPa wind [m s^−1^ (35yr)^−1^] with areas of <90% confidence level masked out. Red contours show the position of rain-belts, defined by the grids where precipitation rate >6.0 mm day^−1^ in the CTRL_CP run. (**b**) Same as (**a**), but for CESM simulated differences between the HEAT_CP run and the CTRL_CP run. (**c**) Shading areas show the linear trend of CVIMF [mm day^−1^ (35yr)^−1^]. Green hatching denotes where the convergence term takes up more than 50% of CVIMF change, and the advection term taking up more than 50% of CVIMF change is marked by black dots. (**d**) The same as (**c**) but for CESM simulated differences between the HEAT_CP run and the CTRL_CP run. This figure was generated by NCL version 6.3.0: http://www.ncl.ucar.edu/.

As seen from the differences between the HEAT_CP run and the CTRL_CP run, overall the model can well capture the “SCS-PhS wetter and southern China drier” pattern when introducing intensified deep convection heating over SCS-PhS in spring (Fig. 3b). Compared to observations (Fig. 3a), biases can also be found; however, they can be well explained as follows.

First, the drying trend over southern China is relatively smaller than that in observation. In the past 35 years, the mid-latitude atmospheric circulation has also changed significantly and the anomalous northerly wind from the mid-latitude continent should also contribute to the drying trend over southern China (Fig. 3a). In our model simulations, the suppressed SRSC can only be attributed to the intensification of low-latitude convection heating, and the mid-latitude effect has not been accounted for in this study.

Secondly, the simulated reinforced precipitation over the Philippine Sea (Fig. 3b) shifts northwestward compared to observation (Fig. 3a), which should be related to two factors. (1) In the CTRL_CP run, the main rain belt over the tropical western Pacific shifts approximately 5° northward and 10° westward. Thus, the location of rainfall response also shifts due to this systematic bias. (2) According to the Gill-type response theory^22^ and the configuration of the HEAT_CP experiment, the anomalous cyclone, which is essential to the convergence in the planetary boundary layer and the ascending motion in the free troposphere, is located to the northwest of the anomalous heating center. Therefore, the anomalous rainfall is situated at the northwestward of the *in-situ* heat forcing center. In spite of the biases, the consistency between observations and the CESM coupled simulations implies that the reinforcement of deep convection heating over SCS-PhS largely contributes to the drying trend over southern China.

Changes in precipitation are mainly controlled by circulation-based moisture transport^18^. Approximately, the convergence of vertically integrated moisture flux (CVIMF) is balanced by precipitation (P) minus evaporation (E). Moreover, the CVIMF can be divided into a moisture convergence (divergence) term dominated by the convergence (divergence) of wind fields and a moisture advection term dominated by heterogeneous moisture field (see Methods). Thus, P minus E reacts to the changes in convergence and advection terms of CVIMF. To understand how the intensified convection heating over SCS-PhS affects SRSC, we calculate the changes in CVIMF and its two components. In both observations and model simulations (Fig. 3c,d), the changes in CVIMF generally illustrate those in precipitation over East Asia and the western Pacific, in which increased (decreased) rainfall is consistent with positive (negative) CVIMF. A closer examination of change in CVIMF suggests some inconsistencies compared to the observed rainfall trends, which is due mainly to the change in the residual term, namely local evaporation (E).

When focusing on the relative contributions of the two individual components to CVIMF, several notable features can be found from observation. Specifically, the change in CVIMF, whether positive or negative, is dominated by the convergence term in low-latitude regions due to the weak moisture gradient in the boundary layer associated with adequate water vapor supply from the ocean surface (Fig. 3c). It is interesting to note that, over the inland southern China, negative CVIMF is primarily attributed to the convergence term, meaning that the anomalous divergence in wind field causes the reduced precipitation. However, over the East China Sea close to costal southern China, it is the advection term that contributes to the negative change in CVIMF. The differences between these two regions indicate different underlying mechanisms that control rainfall changes (Fig. 3c). The fully-coupled model simulations illustrate some valuable clues for the above hypothesis. Although the simulated small CVIMF shifts about 5° northward in southern China, it is mainly limited to inland southern China (Fig. 3d). In model simulations, CVIMF change is dominated by the convergence term over the East Asia and western Pacific regime (Fig. 3d). Indeed, the convergence term makes up more than 80% contribution to the reduced CVIMF over southern China. Thus, we speculate that an underlying rapid and direct dynamic process which is caused by the increased convection heating over SCS-PhS induces an anomalous overturning circulation that suppresses SRSC.

**Figure 4:**
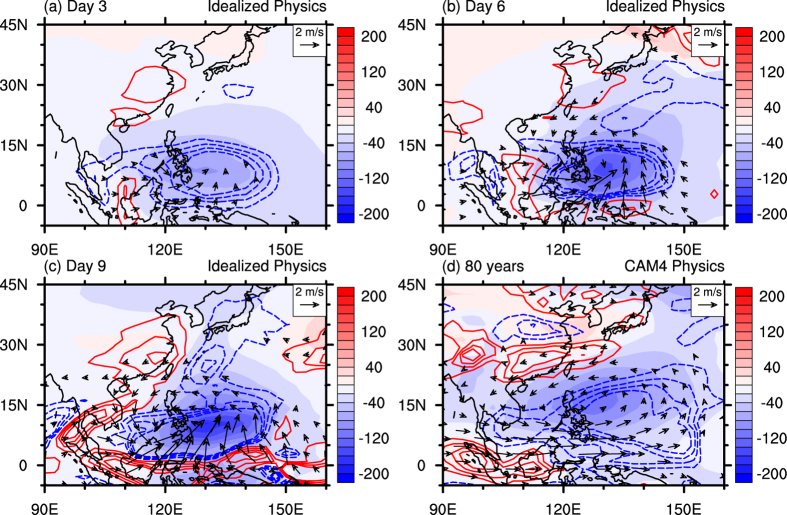
Shading areas show sea level pressure anomalies (Pa). Blue (or red) contours at 0.004 intervals show 500-hPa vertical velocity negative (or positive) anomalies (Pa s^−1^). Vectors show 850-hPa wind anomalies with wind speed smaller than 0.5 m s^−1^ masked out. (**a–c**) Three different snapshots: differences of day 3, day 6, and day 9 between the HEAT_IDEAL run and the CTRL_IDEAL run in the CAM dynamical core integration with idealized physics. (**d**) 80-year mean differences between the HEAT_CP run and the CTRL_CP run in the coupled climate model CESM with CAM4 full physics packages. This figure was generated by NCL version 6.3.0: http://www.ncl.ucar.edu/.

To test the above inference, we further investigate the simulated atmospheric response to the prescribed heating over SCS-PhS in idealized simulations. The idealized model is the dynamical core of the CAM embedded with idealized physics (see Methods for details). Figure 4 shows three snapshots of the idealized integration and an 80-year mean result from the fully-coupled CESM experiments. As demonstrated in Fig. 4a, prescribed heating over SCS-PhS leads to a local ascending motion associated with low-level southerly wind and a decrease in SLP. Note, only after 3 days of integration, descending motion is found over inland southern China. On day 6, anomalous ascending motion strengthens around the heating center, accompanied by a preliminarily-generated low-level anomalous cyclone and a drop in SLP. In the meantime, the downward vertical motion over southern China propagates eastward (Fig. 4b). On day 9, the low-level anomalous cyclone well develops to the northwest of the heating center (Fig. 4c), and upstream anomalies are organized around the heating center and extend poleward over oceans. Importantly, another stronger downstream anomalous center appears near the southeastern coast of China, with broad-scale descending motion over a large part of East Asia after the mature development of the heat-induced circulation. It should be noted that six ensemble members initialized from perturbed climatological conditions also capture this pattern (See Supplementary Information Fig. S4), which implies that the response is robust under the background of springtime atmospheric conditions. The 80-year mean result from the fully-coupled CESM with comprehensive CAM4 physics resembles the response in the idealized simulations, albeit the smoother patterns (Fig. 4d).

The resemblance between the idealized simulations (Fig. 4a–c) and the fully-coupled experiments (Fig. 4d) provides evidence that a heat-induced overturning circulation directly links the springtime rainfalls over SCS-PhS and southern China. More specifically, the enhanced convection heating over SCS-PhS causes local SLP decrease, low-level cyclone, and anomalous ascending motion, while forces descending motion, low-level divergence, and reduced rainfall over southern China. Although modulated by transient flow day by day, anomalous downstream signals pop up one by one over southern China under the background of springtime basic mean flow. This course is a rapid dynamic process, and it not only works within the intraseasonal-interannual time scales, but also contributes to the long-term change between SCS-PhS and southern China.

## Summary and Discussion

In the recent decades, southern China has experienced a significant decreasing trend of precipitation in boreal spring. Meanwhile, precipitation has increased over the SCS-PhS region. Based on observational diagnosis, fully-coupled CGCM simulations, and idealized AGCM experiments, we demonstrate that the enhanced latent heating released by the convection over SCS-PhS leads to suppressed SRSC. Moisture budget analysis indicates that the drying trend over inland southern China is due mainly to the anomalous negative convergence term in CVIMF, which is controlled by a heat-induced overturning circulation from the reinforced convection over SCS-PhS. Further idealized simulations show that the heat-induced overturning circulation, which forces anomalous downstream signals continuously over southern China, can be well established under the circumstance of springtime mean flow. The relationship between the SCS-PhS convection heating and SRSC resembles the P-J teleconnection pattern in summer^25^. For example, in summer, above normal convective activity over the western tropical Pacific tends to be associated with anticyclonic anomalies over the mid-latitudes, giving rise to dry and hot summer days^26^. In boreal spring, although modulated by the springtime mean flow, this teleconnection pattern still works similarly. The convective activity center is to the lower-latitude SCS-PhS, and the downstream anomalies occur in subtropical regions, especially over inland southern China. Therefore, the intensified deep convection heating over SCS-PhS dries southern China in the last 35 years.

Although the spring drying trend over southern China is attributed to the intensification of deep convection over SCS-PhS, it is still not clearly known why the convection has enhanced over SCS-PhS. Accompanied by stronger low-level easterly wind and higher SST in boreal spring (Fig. S2), the atmospheric circulation has changed into a more “La Niña” like pattern over the western equatorial Pacific. Although in the annual mean perspective the long-term change in the Walker circulation is still an open question^27^,^28^,^29^, at least in springtime, the strengthened low-level convergence favors the convection activity over SCS-PhS and in turn enhances the *in-situ* atmospheric latent heating. Undoubtedly, the long-term change in SRSC is a complex issue, and the “SCS-PhS wetter and southern China drier” relationship can only contribute to part of the rainfall decline. What is the role of the changed mid-latitude processes? How will SRSC change in the future and how much can the change be attributed to anthropogenic activities? For instance, recent studies have suggested that increasing aerosols could be one of the major reasons for the decrease in autumn precipitation over mid-eastern China^30^. Serious air pollution events also prevail in springtime, and so what is its contribution to SRSC change? These questions should be addressed by more comprehensive studies.

## Methods

### Graphic software

All figures in this article are produced by the NCAR Command Language (NCL) version 6.3.0, open-sourced software and free to public, by UCAR/NCAR/CISL/TDD, available in http://dx.doi.org/10.5065/D6WD3XH5, while Fig. 1 is additionally paneled by licensed Adobe Illustrator CS6 version 16.0.0.

### Data sets

The SST data is obtained from the Hadley Centre, which provides monthly mean SST and sea-ice concentration in a 1° × 1° spatial resolution. Various types of monthly-mean global precipitation data sets are used in this study. One is the NASA/GSFC Global Precipitation Climatology Project (GPCP) Version 2.2 available since 1979^31^. The Climate Prediction Center merged analysis of precipitation (CMAP)^32^ and Precipitation Reconstruction over Land (PREC/L)^33^ are also used for validations. Monthly atmospheric field data are from the European Centre for Medium-range Weather Forecasts (ECMWF) reanalysis, namely ERA-Interim Reanalysis (1979–2013)^34^. Monthly deep convection heating and atmospheric diabatic heating data (1979–2008) are from the National Centers for Environmental Prediction (NCEP) Climate Forecast System Reanalysis (CFSR)^35^. Given that these 1-hour forecasting variables are mostly determined by the initial data assimilation system, we still consider them as reanalysis data. Daily atmospheric circulation data (1979–2013), used to calculate atmospheric apparent heating Q1 for validation, are from the National Centers for Environmental Prediction/Department of Energy (NCEP-DOE)^36^.

### Model experiments

The NCAR climate model, the Community Earth System Model version 1.2.2 (CESM1.2.2), is a fully-coupled earth system model that can be used for a broad class of scientific problems^37^. We employ both the fully-coupled CESM experiments and idealized simulations to establish the causal relationship between SCS-PhS convection heating forcing and southern China rainfall. We implement the “B_2000 component set” in the coupled experiments. The atmospheric resolution in the CESM CAM4 is 1.9° × 2.5° in longitude-latitude with 26 vertical levels. The oceanic resolution in the CESM POP2 is about 1°. The solar forcing, carbon dioxide, ozone concentration, and aerosol, are all fixed at the levels of year 2000. The idealized model is the finite volume dynamical core of the CESM CAM, embedded by the idealized physics described by Held and Suarez^38^ to ensure that the integration is stable. Horizontal resolution of the model is 1.9° × 2.5° in longitude and latitude.

Model experiment design is summarized in Table 1. In the coupled simulations, the total integration length of CTRL_CP experiment is 280 years, and the output for years 201–280 is analyzed. To distinguish the effect of heating change over SCS-PhS, a sensitive experiment restarted from year 151 of CTRL_CP is performed, in which all external forcings are set to exactly the same as in CTRL_CP but deep convection heating in the domain of 110–155°E/0–15°N is intensified with respect to the observational changes during MAM. The integration period of this experiment, namely HEAT_CP, is from year 151 to year 280, and the output of years 201–280 is analyzed. In the idealized simulations, both the CTRL_IDEAL run and the HEAT_IDEAL run are initialized with the climatological atmospheric condition of the reanalysis for the months of MAM from 1979 to 2013, and the length of integration is 15 days. In the HEAT_IDEAL run, we introduce an additional heating forcing in the domain of 110–155°E/0–15°N. The vertical heating profile is derived from the observational change, and the horizontal distribution of the idealized heating is oval like, with maximum in the domain center which is damped to zero at the boundaries of the domain (See Supplementary Information Fig. S3). To verify the robustness of the response to prescribed heating, ensemble technique is also applied to the idealized simulations with six members in which the initial climatological temperature fields are perturbed randomly by up to 0.1K (See Supplementary Information Fig. S4).

### Q1 analysis

We calculate the change in apparent heating Q_1_ as defined by Yanai *et al.*^39^. For convenience, we define dry static energy as


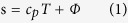


In the equation, s is for dry static energy, c_p_ is the specific heat capacity of air parcel, and Φ denotes geopotential height.

The equation for calculating Q_1_ can be written as


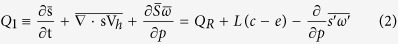


where Q_1_ is apparent heating, consisting of heating due to radiation (Q_R_), release of latent heat by net condensation [L(c-e)], and vertical convergence of the vertical eddy transport of sensible heating 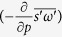. *V*_*h*_ and 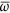 are for horizontal wind and averaged vertical velocity. The horizontal averages are denoted by 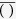. Since the second term on the right hand side of the equation, namely advection term, is a non-linear quantity, we use daily quantity to calculate daily Q_1_ and then determine the monthly mean. In plotting, Q_1_ is divided by c_p_ to show a (K s^−1^) dimension in comparison with other heating data.

### Moisture transport analysis

The moisture budget equation can be written as


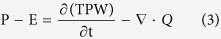


where P is for precipitation and E is for evaporation. TPW is for total precipitable water, and −∇·Q is the convergence of vertically integrated moisture flux (CVIMF). P minus E maintains an approximate balance with the rates of changes in TPW and CVIMF^40^. Although the rate of change in TPW could be obvious over regions like the Tibetan Plateau^41^, for the studied domain, it is safe to assume that the rate of change in TPW is small compared to the change in CVIMF during a long period (See Supplementary Information Fig. 5S). Consequently, CVIMF is approximately balanced by P minus E. Hence CVIMF plays a key role in the hydrological cycle. Following the method provided by Kim and Ha^42^, we divide CVIMF into three terms: a moisture convergence term, a moisture advection term, and an eddy term as shown below:


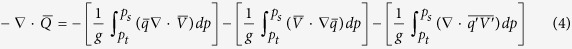


In this study, 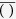 indicates MAM average. The first term on the right hand side of the equation is the moisture convergence term. If the wind is convergent, it commits a positive contribution to CVIMF. The second term is the moisture advection term. If the wind flows from a wet region to a dry region, it shows a positive contribution to CVIMF. The last term is the higher-order eddy term and is not considered in this study, because it makes a relatively much smaller contribution to CVIMF.

## Additional Information

**How to cite this article**: Li, Z. *et al.* Intensified Springtime Deep Convection over the South China Sea and the Philippine Sea Dries Southern China. *Sci. Rep.* **6**, 30470; doi: 10.1038/srep30470 (2016).

## Supplementary Material

Supplementary Information

## Figures and Tables

**Table 1:** Model experiment design.

Experiments	Configurations
CTRL_CP	External forcing at the level of year 2000, 280yr integrated, output from year 201 to year 280 for analyzing
HEAT_CP	Branch run from CTRL_CP year 151 restart fields, with intensified deep convection heating in the SCS-PhS with respect to the observed changes
CTRL_IDEAL	Initiated by MAM mean flow, 15-day integration
HEAT_IDEAL	Same as CTRL_IDEAL, but with prescribed heating profile over the SCS-PhS
