# Supplementary material for: Intensified Springtime Deep Convection over the South China Sea and the Philippine Sea Dries Southern China
Source: Sci Rep. 2016 Jul 27;6:30470. doi: 10.1038/srep30470 (PMC4962042; doi:10.1038/srep30470)
Supplement: Supplementary Information [file srep30470-s1.pdf]

Intensified springtime deep convection over the South China Sea and the Philippine Sea dries southern China

Zhenning Li, Song Yang, Bian He, and Chundi Hu

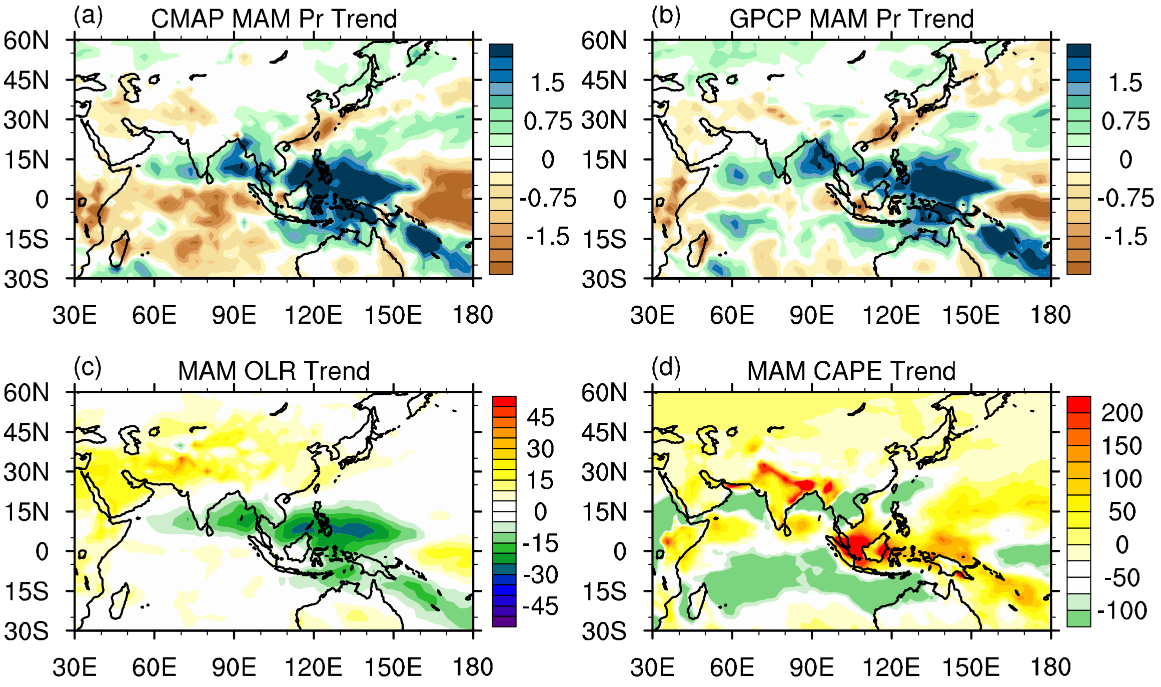

Figure S1. (a) Linear trend of MAM precipitation [mm day^{-1} (35yr)$^{-1}$] for 1979–2013 derived from the CMAP data set; (b) same as (a) but for the GPCP data set; (c) same as (a) but for the MAM OLR [W m^{-2} (35yr)$^{-1}$] from the NOAA OLR data set; (d) same as (a) but for the MAM CAPE [J kg^{-1} (35yr)$^{-1}$] from the ERA-Interim Reanalysis. This figure was generated by NCL version 6.3.0: http://www.ncl.ucar.edu/ .

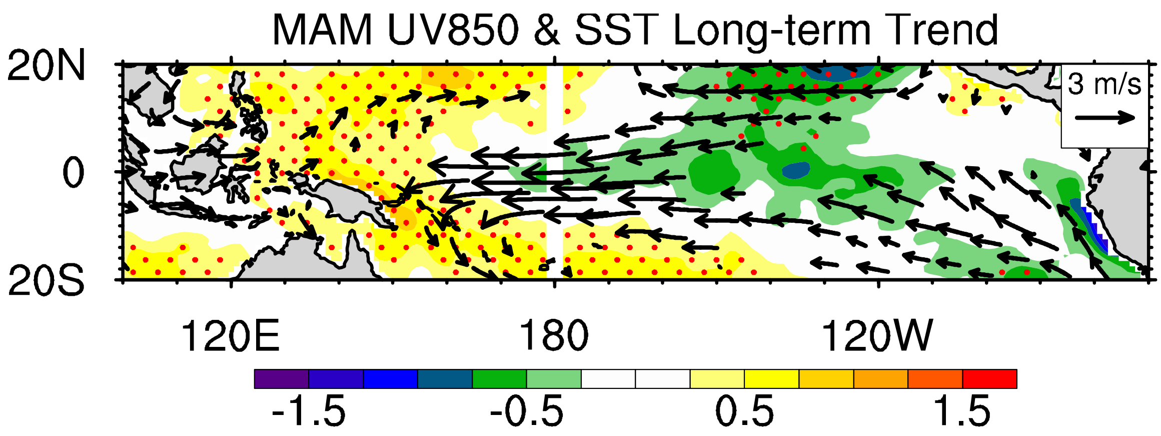

Figure S2. Shading areas show the linear trend of MAM SST [K (35yr)$^{-1}$] for 1979–2013, and red dots denote the values that significantly exceed the 90% confidence level. Vectors denote the linear trend of 850-hPa winds, with areas of <90% confidence level masked out. This figure was generated by NCL version 6.3.0: http://www.ncl.ucar.edu/ .

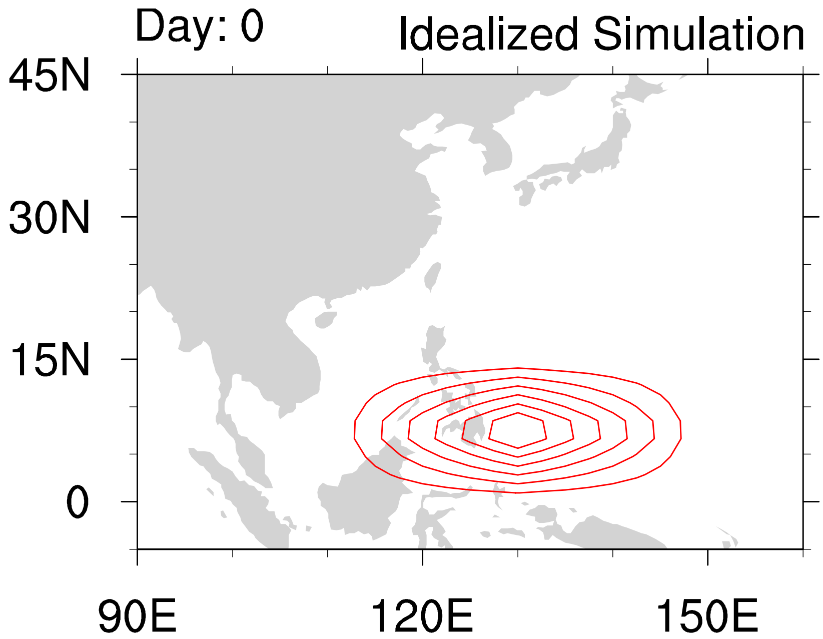

Figure S3. Contours show the distribution of heating rate introduced to the idealized model on the 11th model layer (approximately 450-hPa), with contour intervals of 0.5K/day. This figure was generated by NCL version 6.3.0: http://www.ncl.ucar.edu/ .

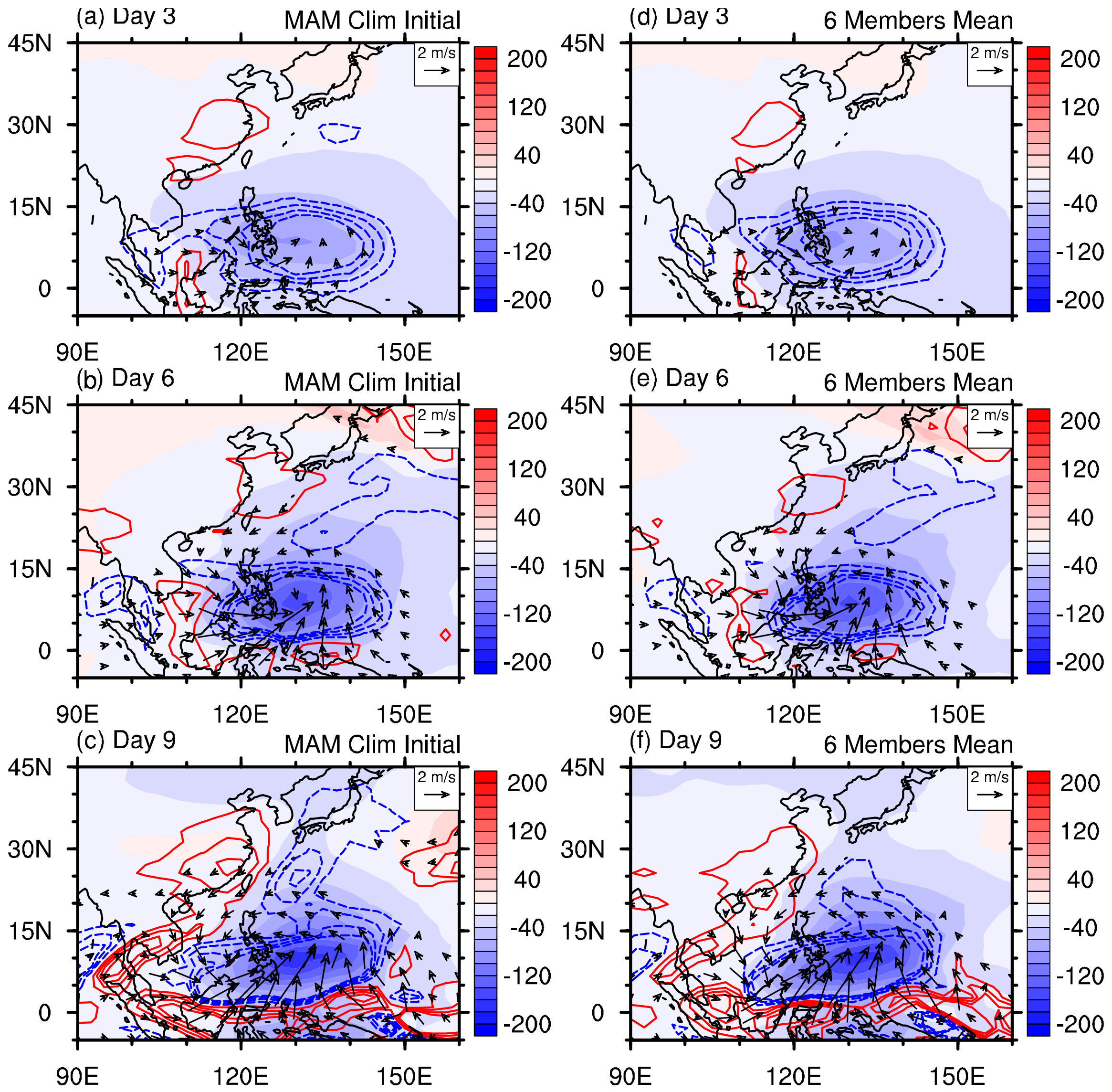

Figure S4. Shading areas show sea level pressure anomalies (Pa). Blue (or red) contours at 0.004 intervals show 500-hPa vertical velocity negative (or positive) anomalies (Pa s^{-1}). Vectors show 850-hPa wind anomalies, with speed smaller than 0.5 m s^{-1} masked out. (a-c) Three different snapshots: differences between the HEAT_IDEAL (initiated by MAM mean flow) run and the CTRL_IDEAL run in the CAM dynamical core integration with idealized physics for of day 3, day 6, and day 9. (d-f) Same as (a-c), but for the differences of 6-member ensemble means, in which the initial temperature fields are perturbed randomly by up to 0.1K. This figure was generated by NCL version 6.3.0: http://www.ncl.ucar.edu/ .

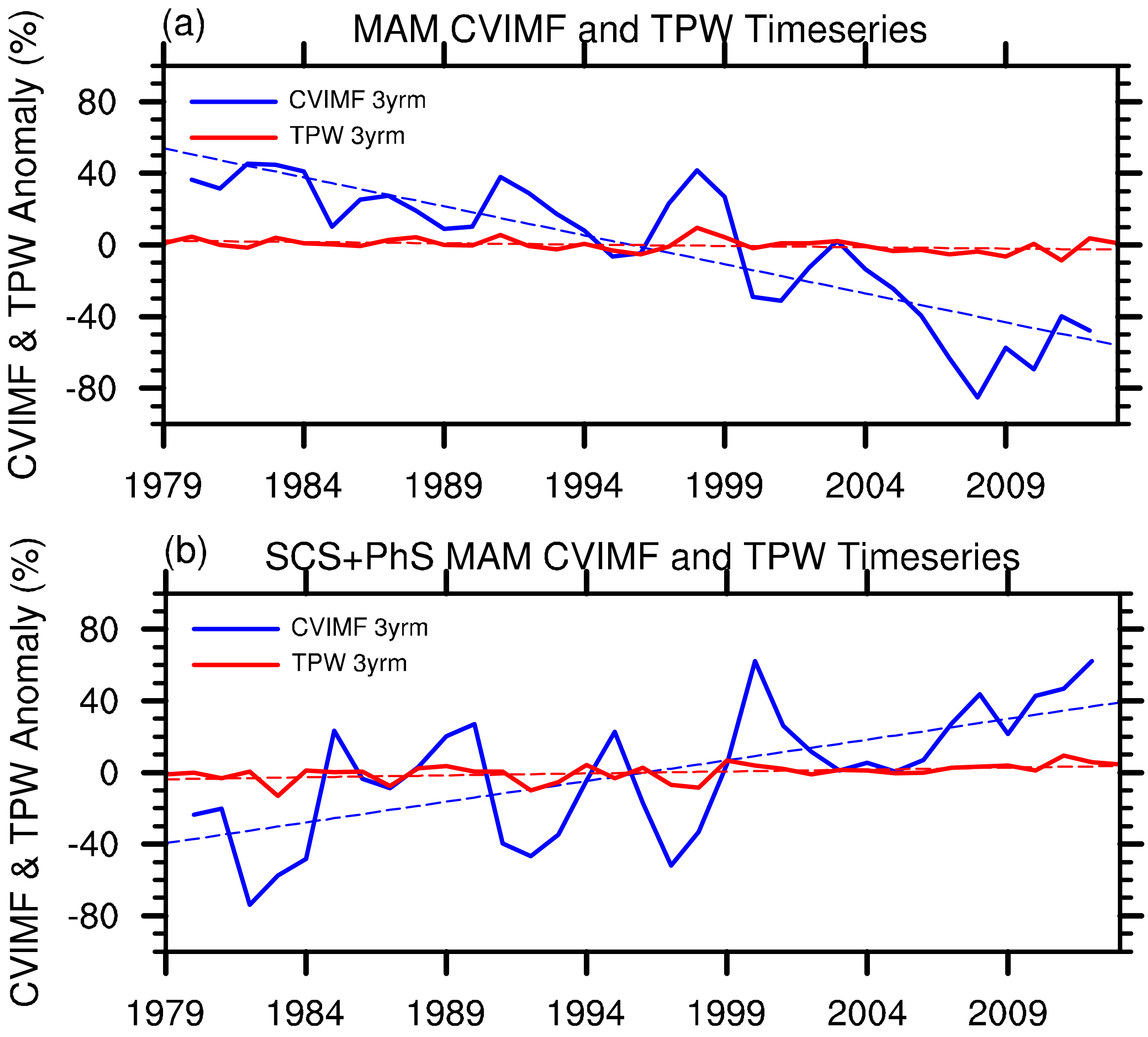

Figure S5. (a) 3-year running means applied on area-weighted MAM mean CVIMF (blue solid line) and TPW (red solid line) anomaly percentages in the upper box in Figure 1a from the ERA-Interim Reanalysis. Blue and red dashed lines are the linear trends of CVIMF and TPW, respectively. (b) Same as (a) but for the lower box in Figure 1a. This figure was generated by NCL version 6.3.0: http://www.ncl.ucar.edu/ .
